# Supplementary figures and images for: Two RNA binding proteins, ADAD2 and RNF17, interact to form a heterogeneous population of novel meiotic germ cell granules with developmentally dependent organelle association
Source: PLoS Genet. 2023 Jul 10;19(7):e1010519. doi: 10.1371/journal.pgen.1010519 (PMC10359003; doi:10.1371/journal.pgen.1010519)

Fig S1

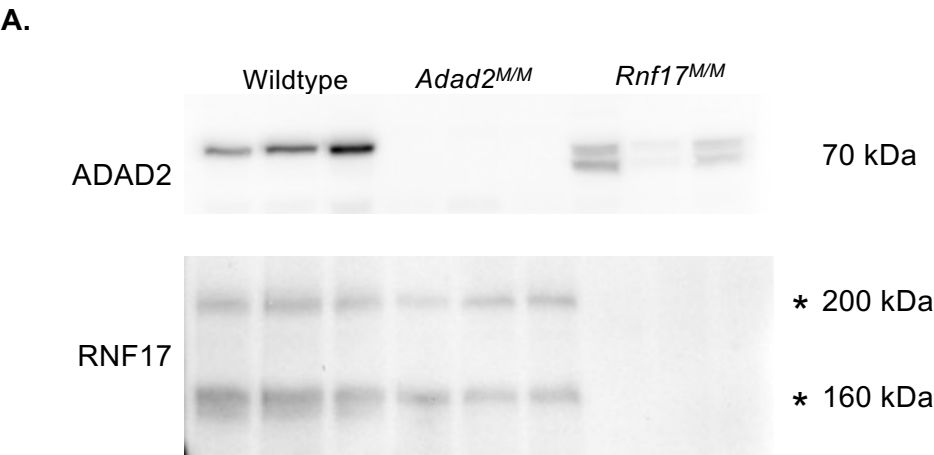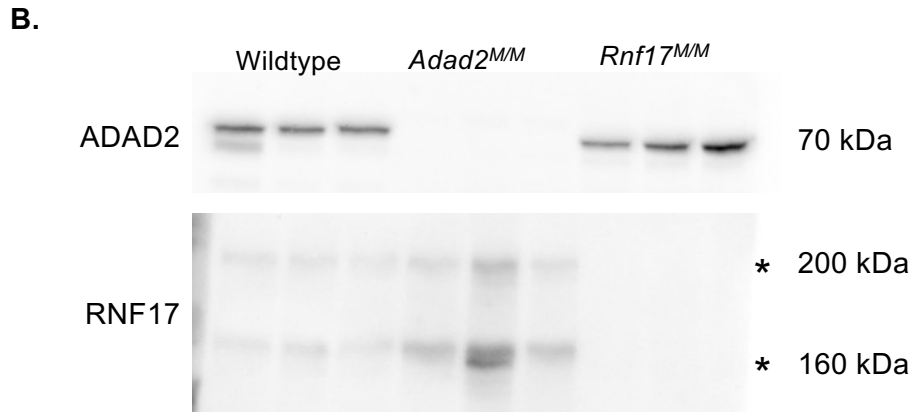

Supplement: S1 Fig — Western blot of ADAD2 or RNF17 in A. 42 dpp wildtype, Adad2M/M, and Rnf17M/M whole testis protein (n = 3) demonstrating complete ADAD2 or RNF17 ablation in the respective genetic model and B. 21 dpp wildtype, Adad2M/M, and Rnf17M/M whole testis protein (n = 3). Asterisks—RNF17 protein isoforms. Approximate molecular weight reported for each band. For loading controls, see S10 Fig. (PDF) [file pgen.1010519.s001.pdf]

Fig S2

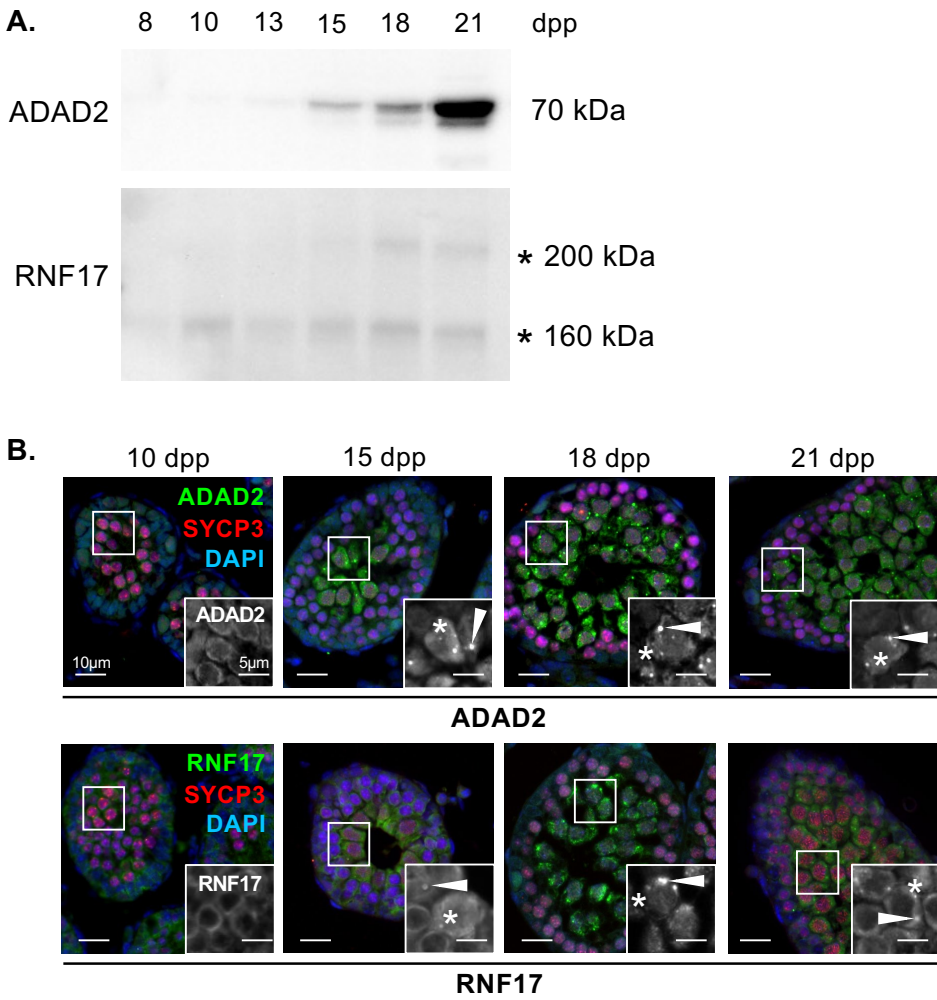

Supplement: S2 Fig — A. Western blot of ADAD2 and RNF17 in wildtype whole testis protein across neonatal and juvenile developmental time points demonstrating the similar developmental profile of ADAD2 and RNF17L. Asterisks—RNF17 protein isoforms. Approximate molecular weight reported for each band. For loading controls, see S10 Fig. B. Immunofluorescence of ADAD2 or RNF17 in wildtype testis across juvenile development. Images represent most mature seminiferous tubule sections at each age and demonstrate large ADAD2 and RNF17 granules forms by 15 dpp. Asterisks–small ADAD2 or RNF17 granules. Arrowheads–large ADAD2 or RNF17 granules. 200x magnification. (PDF) [file pgen.1010519.s002.pdf]

Fig S3

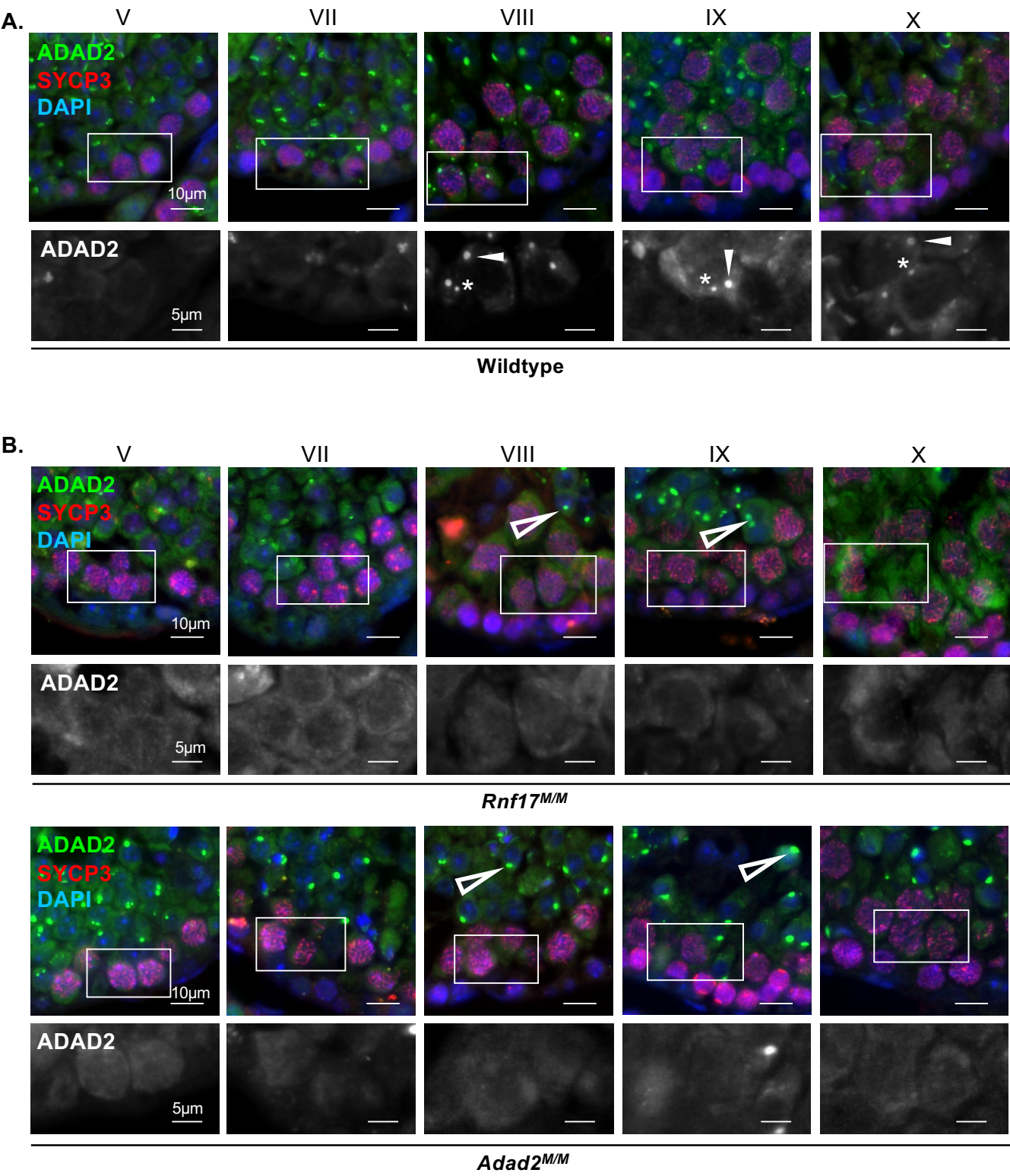

Supplement: S3 Fig — A. Immunofluorescence of ADAD2 across pachytene spermatocyte development in adult wildtype testes demonstrating small and large granule formation in mid-stage pachytene spermatocytes. B. ADAD2 immunofluorescence in Adad2M/M and Rnf17M/M developing pachytene spermatocytes. Note the non-specific ADAD2 signal observed in Adad2 mutant round spermatids. Non-specific spermatid staining marked with an open arrowhead. Roman numerals–testis tubule cross-section stage (V containing early-stage pachytene spermatocytes, VII and VIII containing mid-stage pachytene spermatocytes, IX and X containing late-stage pachytene spermatocytes). Asterisks—small granules and arrowheads—large granules. Red—SYCP3, green—ADAD2, and blue—DAPI. 400x magnification. (PDF) [file pgen.1010519.s003.pdf]

Fig S4

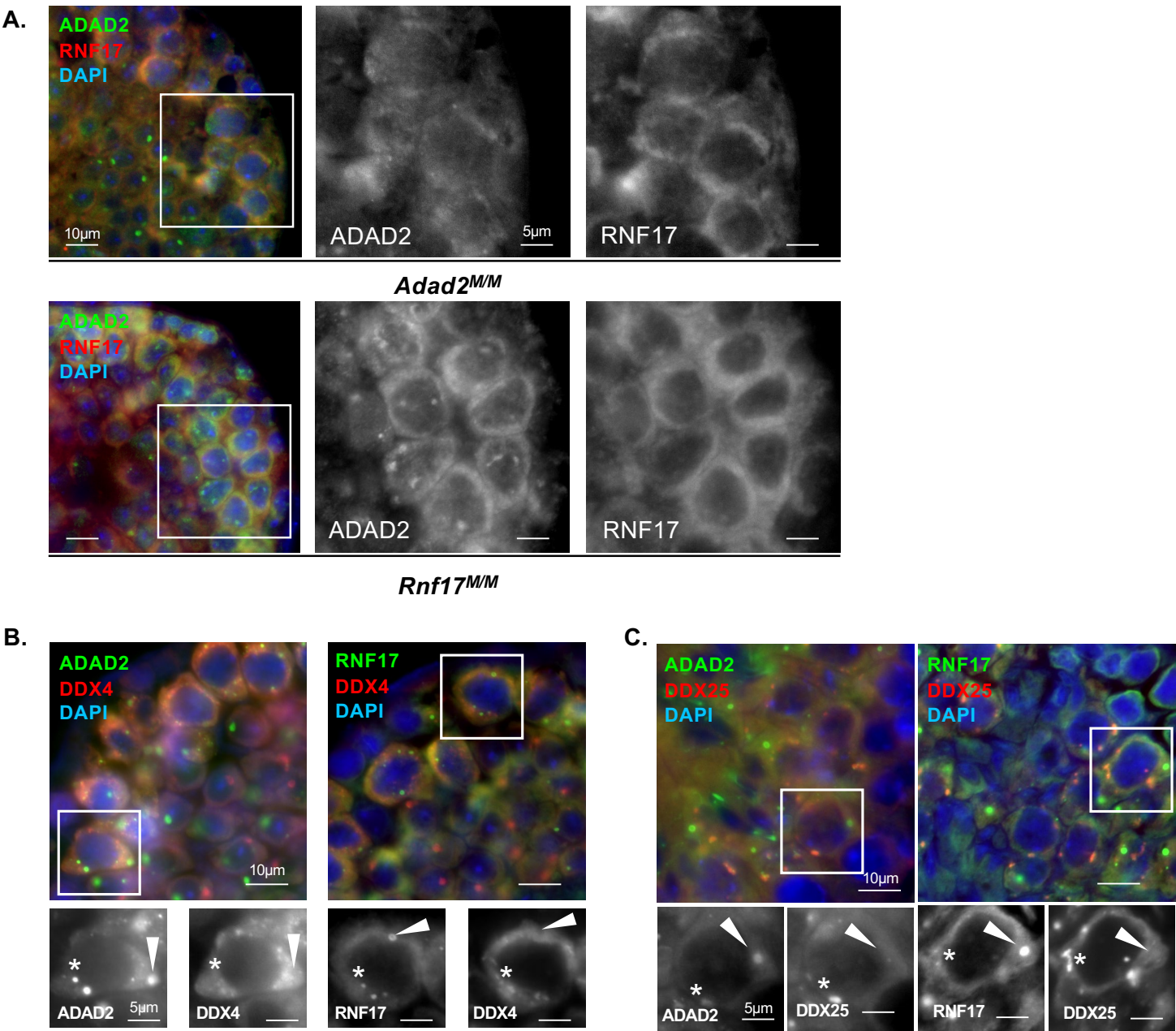

Supplement: S4 Fig — A. Co-immunofluorescence of ADAD2 and RNF17 using fluorophore labeled anti-ADAD2 and anti-RNF17 in Adad2M/M and Rnf17M/M adult testes demonstrating weak, cytoplasmically diffuse non-specific signal. Red–RNF17, green–ADAD2, and blue–DAPI. B. Immunofluorescence of DDX4 and ADAD2 or RNF17 in adult wildtype testes demonstrating neither ADAD2 nor RNF17 colocalize with DDX4. Red—DDX4, green—ADAD2 or RNF17, and blue—DAPI. C. Immunofluorescence against DDX25 and ADAD2 or RNF17 demonstrates that DDX25 colocalizes with some small ADAD2-RNF17 granules but not the large. Red—DDX25, green—RNF17, and blue—DAPI. Asterisks—small granules and arrowheads—large granules. All images 630x magnification. (PDF) [file pgen.1010519.s004.pdf]

Fig S5

A.

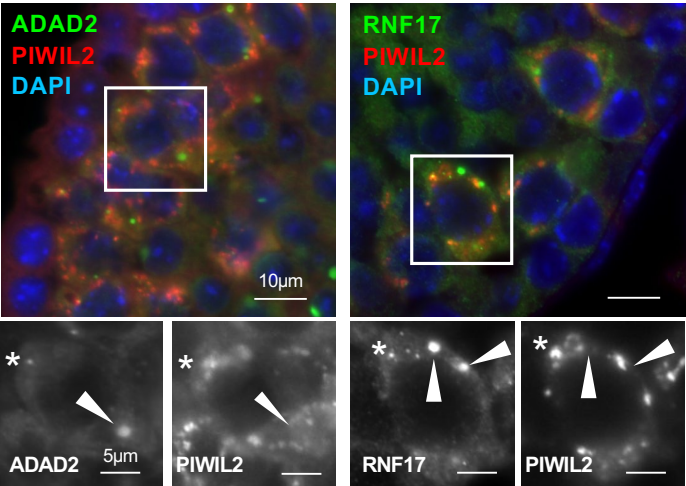

B.

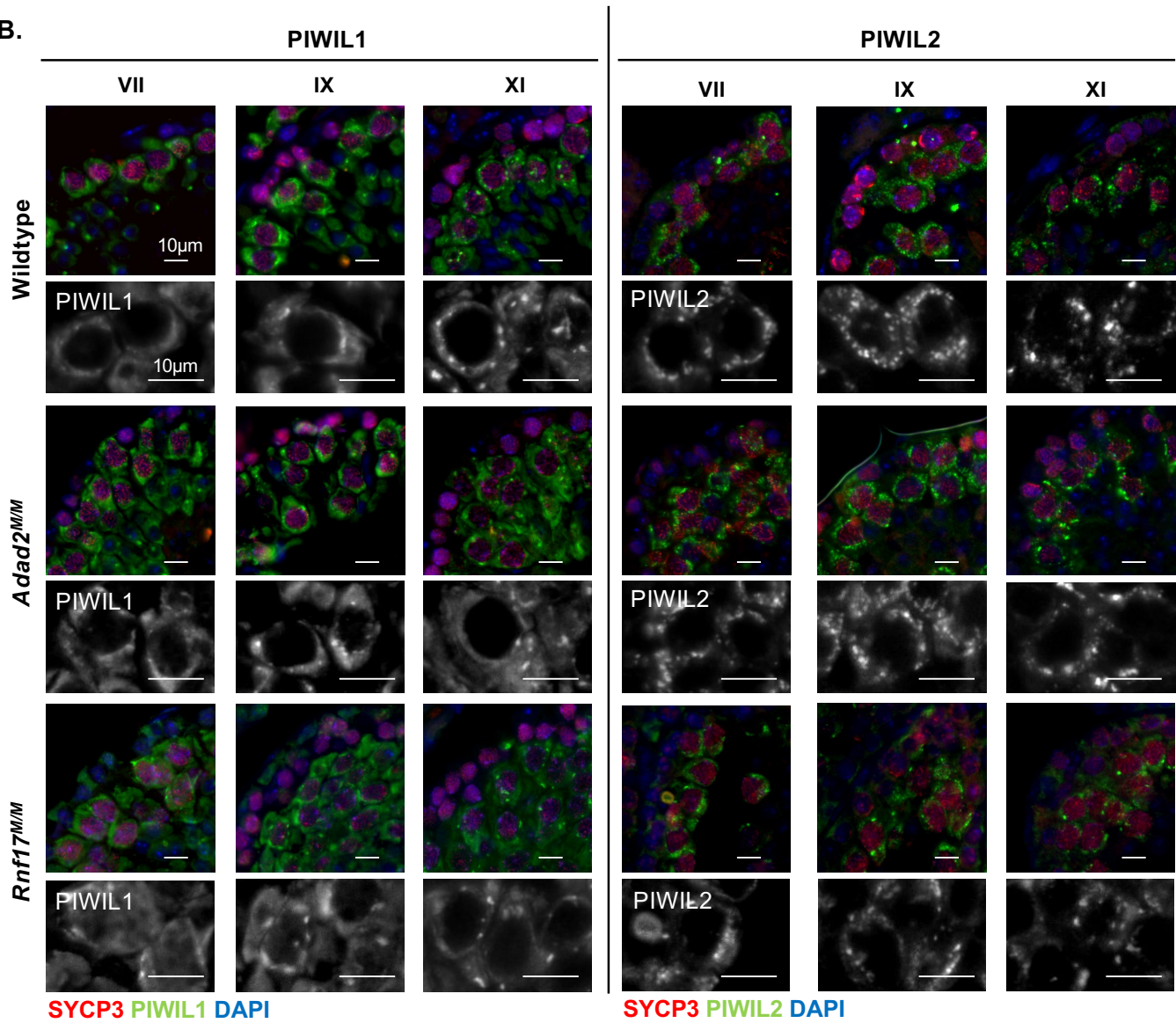

Supplement: S5 Fig — A. Co-immunofluorescence of PIWIL2 with ADAD2 or RNF17 in adult wildtype testes demonstrating a lack of colocalization. Red—PIWIL2, green—ADAD2 or RNF17, and blue–DAPI. Asterisks—small ADAD2 or RNF17 granules. Arrowheads—large ADAD2 or RNF17 granules. 630x magnification. B. Immunofluorescence of PIWIL1 and PIWIL2 in Adad2M/M and Rnf17M/M adult testes by stage (as measured by SYCP3) showing no impact on PIWIL protein localization. Roman numerals–testis tubule cross-section stage. Red–SYCP3, green–PIWIL1 or PIWIL2, and blue–DAPI. 400x magnification. (PDF) [file pgen.1010519.s005.pdf]

Fig S6

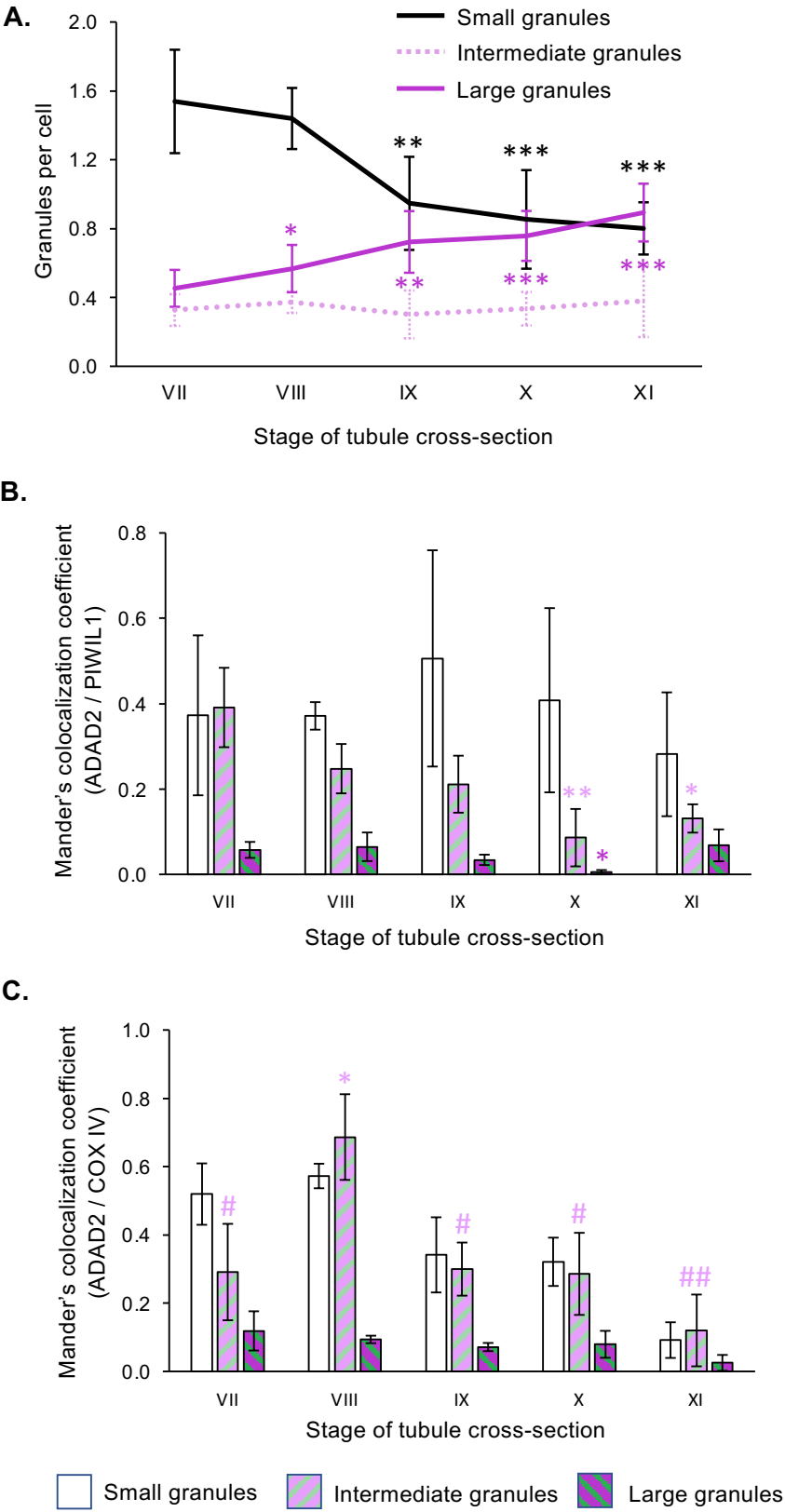

Supplement: S6 Fig — A. ADAD2 granules per cell as a function of tubule cross section stage demonstrating a loss of small granules concurrent with an increase in large. Error bars–standard deviation. Asterisks (black–small granule comparisons, magenta–large granule comparisons)–significant by one-tailed t-test as compared to stage VII, *—p-value < 0.05, **—p-value < 0.001, ***—p-value < 0.0001. Mander’s colocalization coefficients compared by stage and granule size B. ADAD2 localization with PIWL1 (asterisks–significant by two-tailed t-test as compared to stage VII, *—p-value < 0.05, **—p-value < 0.01, ***—p-value < 0.001) and C. ADAD2 localization with COX IV (significance calculated by two-tailed t-test. Asterisk—as compared to stage VII, *—p-value < 0.05. Pound sign–as compared to stage VIII, # < 0.05 and ## < 0.01). (PDF) [file pgen.1010519.s006.pdf]

Fig S7

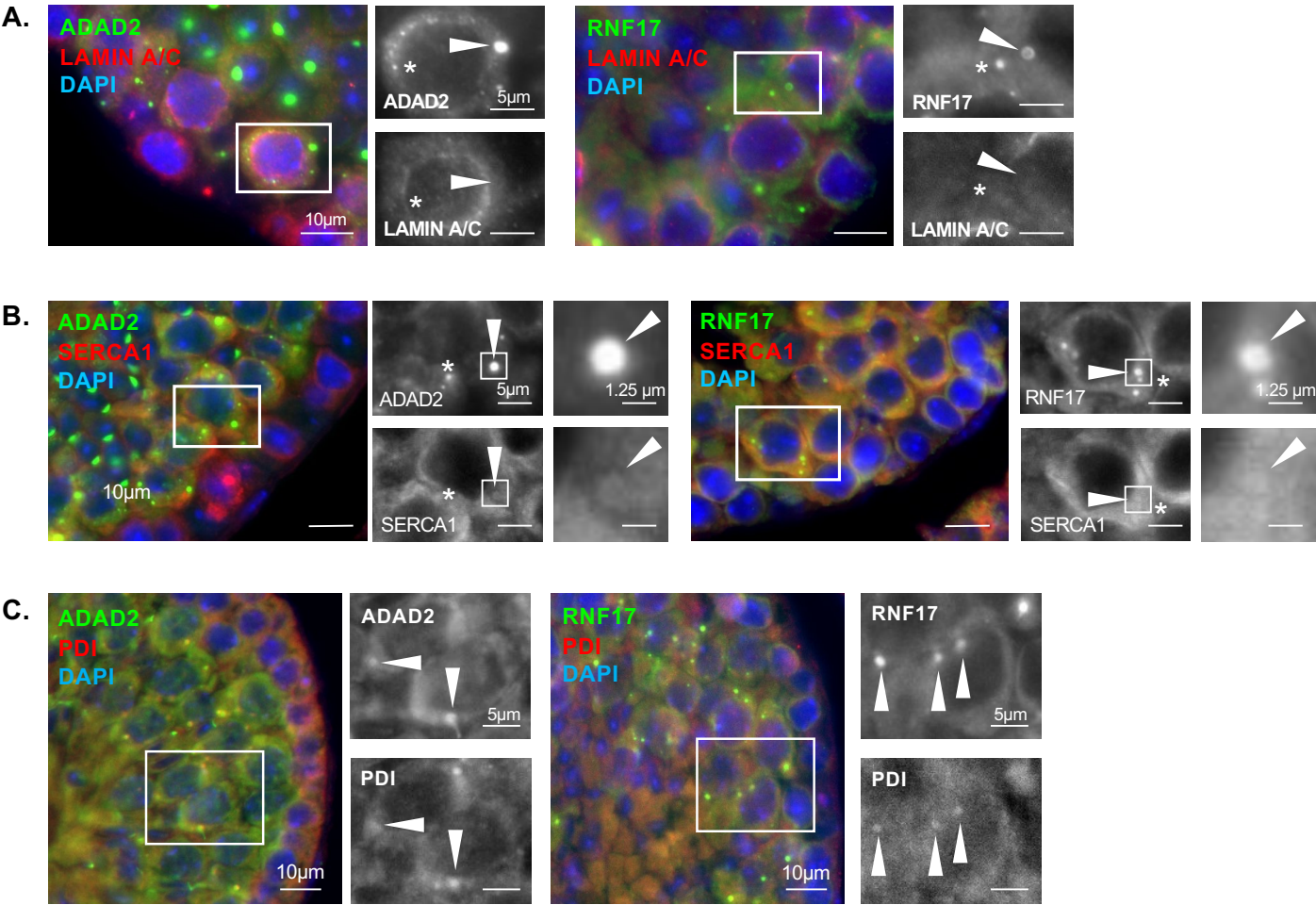

Supplement: S7 Fig — A. Co-immunofluorescence in adult wildtype testes of the nuclear membrane marker Lamin A/C and ADAD2 or RNF17 demonstrating neither ADAD2 nor RNF17 large granules colocalize with the nuclear membrane Red–Lamin A/C, green—ADAD2 or RNF17, and blue—DAPI. 630x magnification for above images. Co-immunofluorescence in adult wildtype testes of the endoplasmic reticulum markers. B. SERCA1 and C. PDI with ADAD2 or RNF17 showing clustering of the ER at large ADAD2 or RNF17 granules. Red–SERCA1 or PDI, green—ADAD2 or RNF17, and blue—DAPI. 400x magnification for B and C. Asterisks—small granules and arrowheads—large granules. (PDF) [file pgen.1010519.s007.pdf]

Fig S8

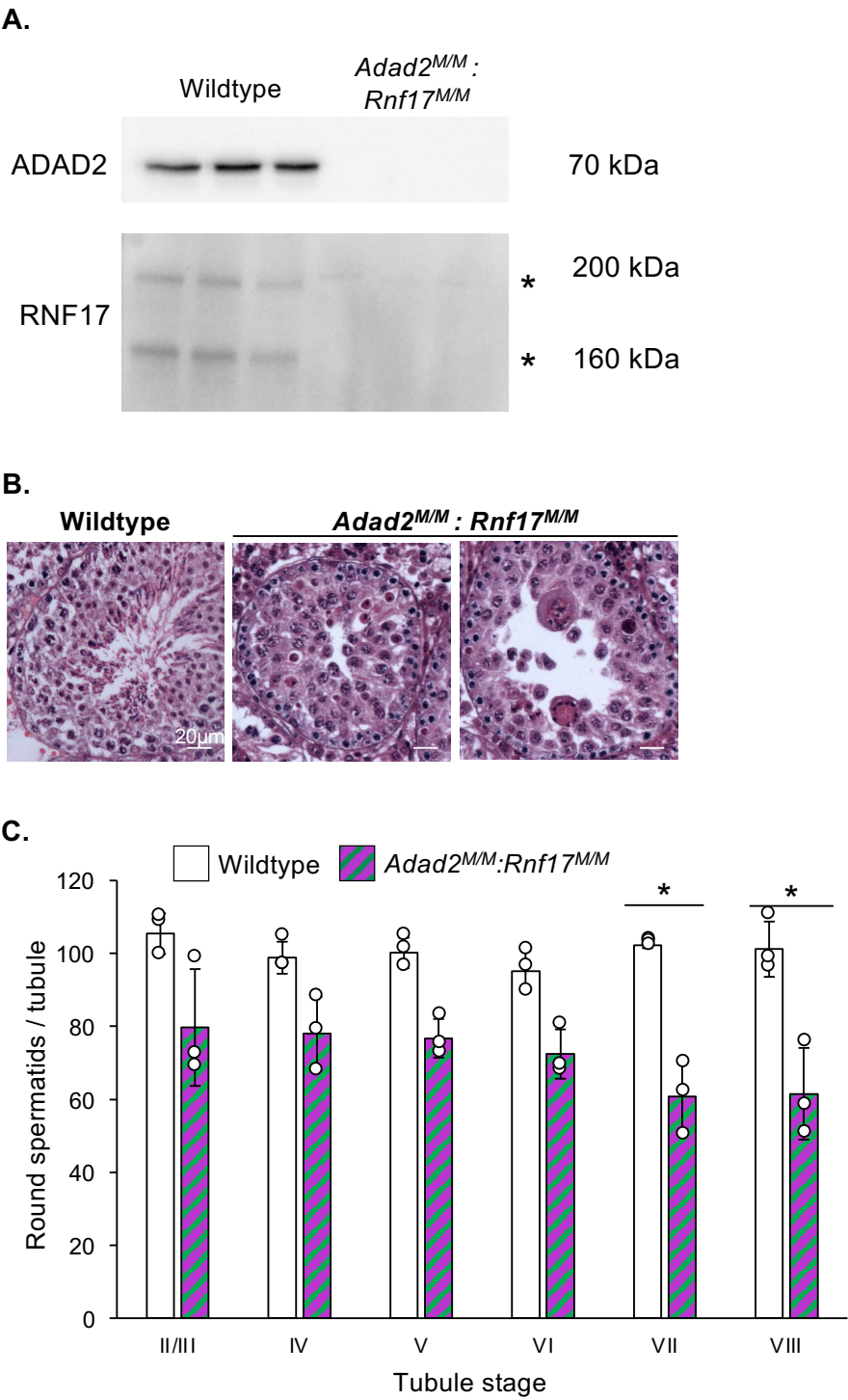

Supplement: S8 Fig — A. Western blot of 21 dpp wildtype and Adad2M/M:Rnf17M/M whole testis lysate (n = 3) probed for ADAD2 and RNF17 confirming ablation of both proteins. Approximate molecular weight reported for each band. B. Adult wildtype and Adad2M/M: Rnf17M/M testis tubule cross-sections stained with H&E demonstrating the range of tubule defects in double mutant testes, including significant post-meiotic germ cell loss. C. Average number of round spermatids per tubule per developmental stage in adult testes from wildtype and Adad2M/M: Rnf17M/M animals (n = 3). Data are mean ± s.d. Significance was calculated using an unpaired, one-tailed Student’s t-test (*P < 0.05, **P < 0.005, ***P < 0.0005). (PDF) [file pgen.1010519.s008.pdf]

Fig S9

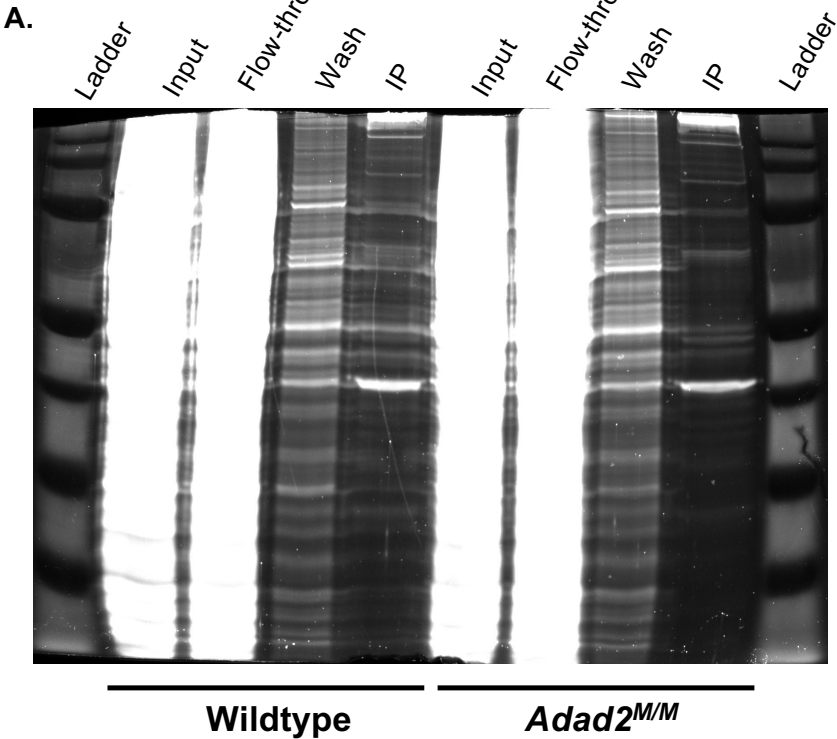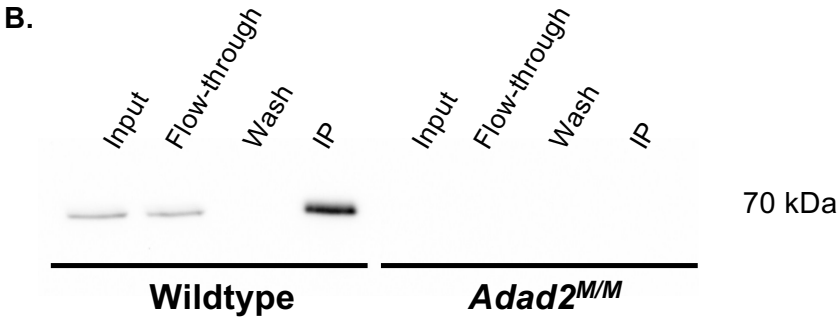

Supplement: S9 Fig — A. SYPRO-Ruby stained SDS-PAGE gel. B. Western blot against ADAD2. Approximate molecular weight reported for each band. (PDF) [file pgen.1010519.s009.pdf]

Fig S10

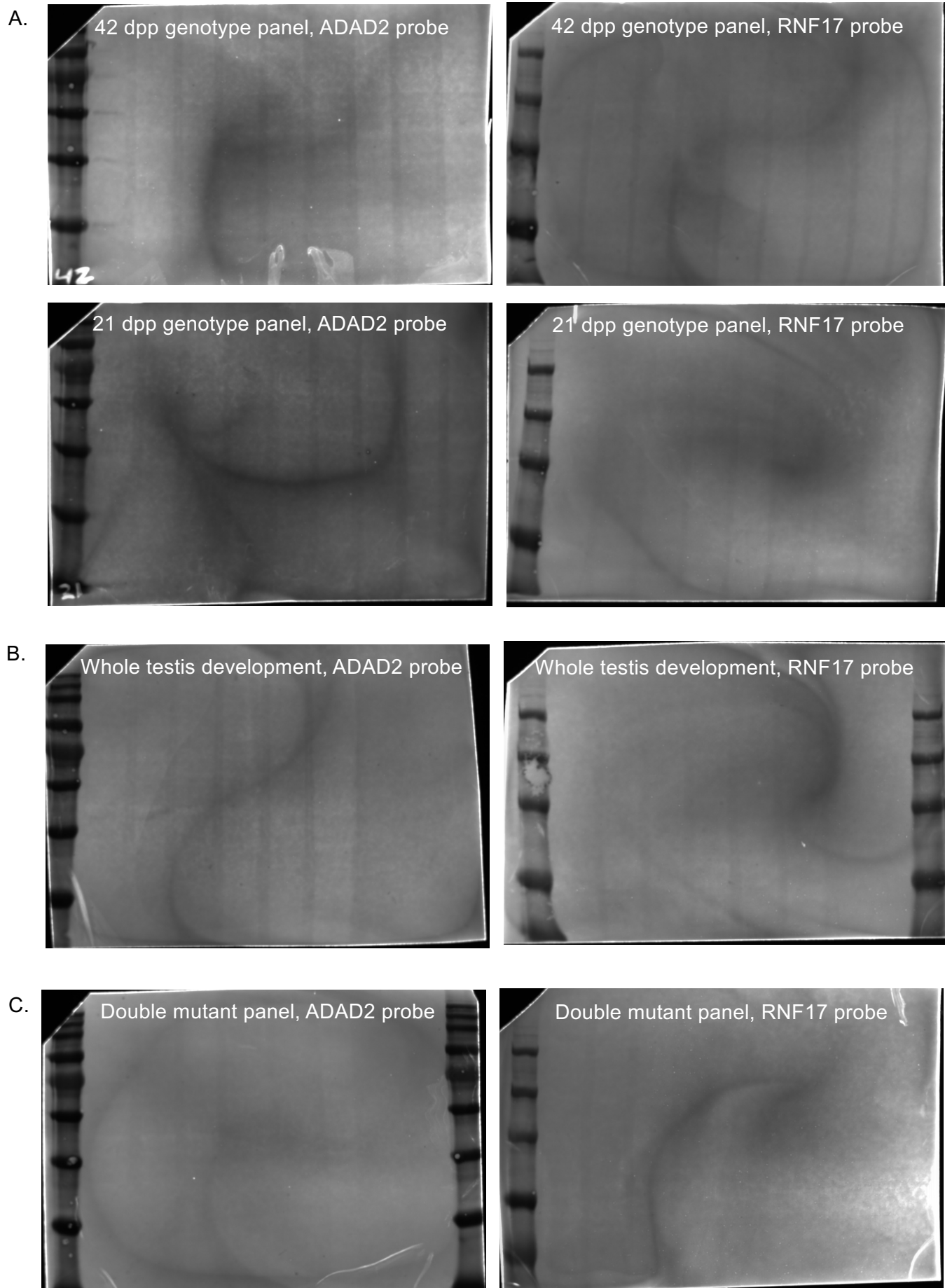

Supplement: S10 Fig — SYPRO-Ruby stained membranes for blots shown in A. S1 Fig, B. S2 Fig, and C. S8 Fig showing equal loading across lanes. (PDF) [file pgen.1010519.s010.pdf]
