# Supplementary material for: Two RNA binding proteins, ADAD2 and RNF17, interact to form a heterogeneous population of novel meiotic germ cell granules with developmentally dependent organelle association
Source: PLoS Genet. 2023 Jul 10;19(7):e1010519. doi: 10.1371/journal.pgen.1010519 (PMC10359003; doi:10.1371/journal.pgen.1010519)
Supplement: Attachment — Submitted filename: Response to reviewers.pdf [file pgen.1010519.s016.pdf]

To whom it may concern,

The authors would like to thank the reviewers for their thoughtful commentary and helpful suggestions, which we feel have significantly improved this manuscript. As a response to the reviews, we have made extensive changes to the manuscript including shifting our focus to the large ADAD2 granule and how it forms. As suggested, we have further delved into the potential mechanisms of action for ADAD2 by more carefully defining the granule populations and their composition. Further, we have explored the dynamic relationship between the various ADAD2-RNF17 granule populations and propose a model of their action. This new data leads to a holistic model of ADAD2 function and reveals a potentially conserved regulatory mechanism between germ cell granules and other RNA granules. Responses to individual comments may be found below in blue.

Sincerely,

The Authors

Reviewer #1: The study by Chukrallah et al. investigates the molecular composition and function of the cytoplasmic ribonucleoprotein granules, or germ granules, in meiotic cells. Electron microcopy and immunostaining analyses have revealed distinct type of granules in spermatocytes, and many of these granule types remain poorly characterized. It has been shown before that the Adenosine deaminase domain-containing protein 2, ADAD2, specifically localizes to the cytoplasmic granules meiotic germ cells, and that ADAD2 is required for haploid male germ cell differentiation. Here the authors show that ADAD2-granules are also positive for RNF17, a Tudor domain-containing protein implicated in the PIWI-interacting RNA (piRNA) pathway. ADAD2 and RNF17 also interacted in the testis, and the mutant mice for Adad2 and Rnf17 were demonstrated to share very similar spermatogenic phenotype. The interaction was important for the germ granule localization, and ADAD2 failed to localize to the granules in the absence of RNF17 and vice versa. The authors also showed that there is heterogeneity in ADAD2-RNF17 positive granules with some of them co-localizing with PIWI proteins, while others co-localizing with a translational regulator NANOS1 to cup-shaped structures associated with the endoplasmic reticulum.

Although the study provides interesting information about the different germ granules in spermatocytes, it remains descriptive, mostly concentrating on characterizing the protein localizations without novel mechanistic data on the germ granules or ADAD2 functions. Both mutant mouse lines have already been published in earlier studies, and the granular cytoplasmic localization of ADAD2 and RNF17 have also been published, including the observation that ADAD2 granules do not overlap with DDX25. It is an interesting result that ADAD2 and the Tudor protein RNF17 co-localize to the granules distinct from the IMC or CB, and appear to regulate the similar phase of spermatogenesis. However, without further functional characterization the study remains quite thin, and it remains unclear, for example, if ADAD2 is involved in the piRNA pathway, e.g. in the suppression of the ping-pong amplification, a process that has been shown to be regulated by RNF17.

We appreciate Reviewer #1's concern regarding lack of mechanistic insight. To the question of piRNA pathway impacts, we would like to direct the reviewer to two reports published during the review and revision phase of this manuscript describing the influence of

ADAD2 loss on piRNA biogenesis (Xiong et al. 2023; Lu et al. 2023). Both find that ADAD2 has a similar impact on piRNA production as RNF17. Unfortunately, neither paper distinguishes between the two granule populations, in particular their protein composition, with the exception of noting a later appearing granule population is negative for PIWIL2 ((Xiong et al. 2023)). These findings are consistent with our observations but don't address the function of the PIWIL2 negative granule (in our work, the large granule). Secondly, neither report discusses the distinct phenotypic impact of *Rnf17* mutation on heterochromatin which provides valuable insight into RNF17 function and has been entirely undescribed until this work.

In order to address these questions, we have focused this work on defining the mode of formation and composition of the large granule. New analyses toward this aim are described in **Figures 5, 7, and 8**; **Supp Figures 5, 6, and 7**; and the new Results sections titled "*The molecular composition and organelle association of the ADAD2-RNF17 granule changes across germ cell development*", "*The large ADAD2-RNF17 granule is a P-body that requires both proteins to form*", and "*Translation regulators localize to distinct regions of the large ADAD2-RNF17 granule*"; as well as the highly modified Results section titled "*The large ADAD2-RNF17 granule is associated with the endoplasmic reticulum*". They demonstrate the large granule contains numerous translation regulators, has distinct protein domains, and associates closely with endoplasmic reticulum cisternae enriched for the protein folding chaperone PDI. Combining our current analyses with previously published analyses of *Adad2* mutants (Chukrallah et al. 2022) , we conclude this second granule is likely a site of translation regulation in the meiotic male germ cell. Further, this new data describes the temporal appearance of the large granule. Importantly, this data suggests the granule populations described here represent a continuum of maturation and may be regulated by their interaction with the ER. This insight informs on both the biology of the ADAD2 granule as well as several outstanding questions in the broader RNA granule field.

Specific comments:

- The title states that the ADAD2/RNF17 germ granules are required for normal male fertility. These proteins do co-localize to the same cytoplasmic granules, but they also localize elsewhere – therefore, it feels premature to say that these granules are required for male fertility, and I think it can only be concluded that ADAD2 and RNF17 are required for male fertility.

After careful consideration, we agree with this reviewer's statement. To that end, we have changed the title of this work to *"Two RNA binding proteins, ADAD2 and RNF17, interact to form a heterogeneous population of novel meiotic germ cell granules with developmentally dependent organelle association"*. We have further modified the text throughout to make this distinction clear.

- Abstract: the exact point of the following sentence is difficult to understand: "Lastly, a double Adad2-Rnf17 mutant model demonstrated loss of ADAD2-RNF17 granules themselves, as opposed to loss of either ADAD2 or RNF17, is the likely driver of the Adad2 and Rnf17 mutant phenotypes." Should be clarified. Can you really make a conclusion that the loss of ADAD2-RNF17 granules causes the phenotype, is it also possible that the loss of ADAD2-RNF17 interplay in general, inside or outside the granules, causes the phenotype?

We appreciate this concern and agree. To that end, we have clarified that the loss of the ADAD2 and RNF17 interaction is the likely driver of the individual phenotypes. We have edited the abstract as well as the results and discussion to reflect this.

Have you studied the granules containing ADAD2 and RNF17 by electron microscopy in the mutant germ cells, do you know if the whole granule structure is disrupted, or if the granules are still there even in the absence of these proteins?

We very much appreciate this line of questioning. Unfortunately, EM as a method is currently unavailable to us. As an alternative approach, we expanded experiments focused on spermatocyte granule composition in the context of either ADAD2 or RNF17 loss. As identification of the ADAD2-RNF17 granule without one or the other is impossible, we first sought to define additional components of the granule. These include NANOS1, PUM1, and the P-body marker EDC3. Analysis of EDC3 localization in both mutants demonstrated abnormal or failed EDC3 granularization in both. Although not conclusively demonstrating the granule itself fails to form, lack of three key components (ADAD2, RNF17, and EDC3) would suggest that if an aggregate forms of a similar ultrastructure, it would not be of the same nature as the intact granule. These results are described in detail in the Results section titled "*The large ADAD2-RNF17 granule is a P-body that requires both proteins to form*" and the newly added **Figure 5**.

- line 55: spell out "piRNA" when it appears for the first time
- line 55-56: piRNAs have also important role in transposon silencing, this function should be mentioned.
- line 57: maybe would be better to say "the primary piRNA-binding proteins" rather than "biogenesis factors". Many other important factors are involved in the piRNA biogenesis, and PIWI protein also mediate piRNA functions after the biogenesis.
- line 72: spell out ADAD2

Thank you for these suggestions. They have all been addressed in the highly modified manuscript.

- Western blot images: The molecular weight marker sizes have to be shown in western blots.

Throughout, molecular weights have been added to western blots.

Please also explain what are the samples (Input Flow-through, Wash, IP) in the figure legend of Fig. 1C.

This information has been added to the figure legend.

- The mass spec results should be provided as a supplementary file, including the information about the peptide hits in each sample.

This data has been provided as **Supplemental Table 1.**

- Fig. 3A is missing the information on which antibodies were used in the immunostaining.

This information has been added to the figure.

- Fig. 4B,C: Co-localization of ADAD2 and RNF17 with PIWIL1 and PIWIL2 is somewhat difficult to interpret. To me it looks like ADAD2/RNF17 and PIWIL2 granules are distinct, and ADAD2/RNF17 would not co-localize with PIWIL2 to same granules, but

ADAD2/RNF17 and PIWIL2 granules are just found often very close to each other?

Thank you for pointing this out. After more careful evaluation, it does appear that PIWIL2 does not co-localize with ADAD2 or RNF17. We have corrected the text to better reflect this. Importantly, we very much appreciate this observation as it led us to more carefully delineate the relationship between the small A2-R17 granule and the IMC, please see newly added section "*Small ADAD2-RNF17 granules are a mix of IMC-associated and cytoplasmic aggregates*".

- Fig. 6A: what is the strong ADAD2-positive structure in round spermatids? Does the antibody give (unspecific?) acrosomal staining?

The ADAD2-positive structure in round spermatids is nonspecific background staining as evidenced by its presence in *Adad2* mice. This has been highlighted in **Supplemental Figure 3B** (open arrowheads) and included in the figure legend**.**

Reviewer #2: In this manuscript, the authors identified RNF17 as a major ADAD2 binding partner and together forming a novel type of germ granule in meiotic male germ cells. Through a meticulous study of ADAD2 and RNF17 positive germ cell granules by imaging and mouse genetic models, they show the dynamic formation of subtypes of granules and potential functional involvement in germ cell development. Germ granule dynamics are understudied in meiotic cells. This study paves the way for understanding new connections of germ granules to different cellular pathways critical for germ cell differentiation.

Major comments:
The round spermatid chromocenter defect in Adad2 and RNF17 KO mice is intriguing. How would the authors explain RBPs like ADAD2 and RNF17 affect heterochromatin formation in the nucleus. Is there a genetic link between ADAD2/RNF17 and chromocenter regulators such as TLF and BRDT [ref 27, 28]?

Thank you for this interesting question! There is no known genetic link between TLF or BRDT and either ADAD2 or RNF17. However, they share an extremely rare phenotype suggesting they modulate a similar pathway. Unlike TLF and BRDT, which are direct regulators of heterochromatin, this and previous work (Chukrallah et al. 2022) suggests that ADAD2 (and likely RNF17 by association) influences heterochromatin indirectly, via transcript-specific translation regulation. We have expanded on the notion that ADAD2 is a translation regulator by identifying PUM1 as a component of the large ADAD2-RNF17 granule (see newly added Results section "*Translation regulators localize to distinct regions of the large ADAD2-RNF17 granule*") and by identifying PDI, a protein folding chaperone, as closely associated with the granule (see newly added data in the Result section "*The large ADAD2-RNF17 granule is associated with the endoplasmic reticulum*"). Statements regarding the function of the large ADAD2-RNF17 granule and its relationship to heterochromatin has also been added to the discussion.

Line 215: The authors stated that previous analysis of ADAD2 granules has demonstrated ADAD2 does not colocalize with DDX25 [23]. But in fact, reference [23] indicates that ADAD2 granules showed partial overlap with DDX25 in late pachytene and diplotene spermatocytes (Fig. 5C of Elizabeth Snyder et al. 2020 [23]). The authors need to add an image of ADAD2 and DDX25 co-staining in late pachytene or diplotene (stage

IX-XI). In Fig S4C, there are some granules with yellow-orange color (overlap of green and red) which means that RNF17 and DDX25 partially overlap.

We very much appreciate this suggestion as it caused us to go back and re-evaluate the DDX25 data presented in the original submission. We agree with Reviewer #2 that there is limited overlap between ADAD2, and RNF17, and DDX25. Notably, this overlap is exclusive to a subpopulation of small ADAD2-RNF17 granules and is another example of heterogeneity in the population. We have included new images to reflect this in **Supplemental Figure 4C** and wording to this effect in the relevant results section.

Different types of granules are still vaguely defined. Granules are in a dynamic state of aggregation and dissociation. Some groups also defined granules in late pachytene and diplotene spermatocytes as chromatoid body precursors. Without immune-EM, it is still not convincing to conclude that large ADAD2/RNF17 granules are the so-called cluster of 30 nm particles.

We entirely agree with Reviewer #2 on this. For this reason, we set out to carefully define the differences and potential relationships between the various populations of ADAD2-RNF17 granules. These analyses revealed a signature of granule maturation that fits very nicely with the dynamic granule model. These analyses are included in the new Results section "“*The molecular composition and organelle association of the ADAD2-RNF17 granule changes across germ cell development*” as well as **Supplemental Figure 6**. They are further summarized in **Figure 10**. Additionally, we have included in the Discussion a paragraph highlighting the dynamic nature of the ADAD2-RNF17 granules and other literature suggesting the dynamic nature of the other granules. Based on these findings, we agree it is premature to assign the large granule as the cluster of 30 nm particles. To that end, we have edited the relevant sections in the results section and removed that element of the discussion.

To the question of the chromatoid body precursor, most groups have defined the precursor as containing DDX25 and DDX4. As such, the large granule is definitively not the chromatoid body precursor (DDX25 and DDX4 negative), nor are the small granules (DDX4 negative with some DDX25 positive). However, given evidence herein and evidence from others, we cannot preclude the possibility that the large ADAD2-RNF17 granule and the CB precursor share components nor that a subpopulation of the small granules are remodeled and give rise to the CB precursor. Mention of this can be found in the Discussion.

What reason could the ADAD2-RNF17 granule be interacting with the ER? Dive further in this in the discussion would be helpful.

Thank you for bringing up this key point, which has been a source of active discussion in our group for many months. The ER has long been known to be a ribosome-rich site of translation, specifically for secreted proteins. However, modern analyses have determined ER-bound ribosomes interact with a huge portion of the transcriptome and facilitate the translation of secreted as well as cytosolic and organelle-bound proteins. As a result, it seems feasible the ADAD2-RNF17 granule associates with the ER to facilitate translation. From a granule perspective, an even more exciting clue comes from very recent studies of stress granules, which were found to associate with and be structurally regulated by the three-way junctions of ER cisternae. Thus, the ADAD2-RNF17 granule may be associating with the ER to facilitate dynamic changes in shape or composition and ER regulation may be a conserved mechanism across multiple granule types. Statements to this effect have been added to the discussion.

Minor comments:
Line 113, Line 125: in vitro should be in vivo.

The above lines describe experiments performed ex vivo. Although our immunoprecipitation protocol does leverage protein lysates derived from whole tissue, the interaction itself occurs in vitro, thus the selected usage.

Fig S1 and Fig S2A: These WB images need to have internal control such as β-ACTIN or GAPDH to ensure consistent sample loading.

Due to the disparity in molecular weight between ADAD2 (70 kDa) and RNF17 (160 and 200 kDa), finding a reliable control for re-probing blots is difficult. To that end, we use SYPRO Ruby stain as a total protein loading control. These images can be found in **Supplemental Figure 10**. Language directing the reader to these controls has been added to the text.

Fig 3A: the authors should label RNF17, SYCP3 and DAPI on the image.

This has been corrected.

Typos:
Line 143: add comma after "similar profile"
Line 298: change "therefor" to "therefore"
Line 461: "Adad2:Rn17" should read "Adad2:Rnf17".

All the listed typos have been corrected.

Literature Cited

Chukrallah, Lauren G., Aditi Badrinath, Gabrielle G. Vittor, and Elizabeth M. Snyder. 2022. "ADAD2 Regulates Heterochromatin in Meiotic and Post-Meiotic Male Germ Cells via Translation of MDC1." *Journal of Cell Science* 135 (4): jcs259196. https://doi.org/10.1242/jcs.259196.

Lu, Yonggang, Ippei Nagamori, Hisato Kobayashi, Kanako Kojima-Kita, Kenjiro Shirane, Hsin-Yi Chang, Toru Nishimura, et al. 2023. "ADAD2 Functions in Spermiogenesis and PiRNA Biogenesis in Mice." *Andrology* 11 (4): 698–709. https://doi.org/10.1111/andr.13400.

Xiong, Mengneng, Lisha Yin, Yiqian Gui, Chunyu Lv, Xixiang Ma, Shuangshuang Guo, Yanqing Wu, et al. 2023. "ADAD2 Interacts with RNF17 in P-Bodies to Repress the Ping-Pong Cycle in Pachytene PiRNA Biogenesis." *Journal of Cell Biology* 222 (5): e202206067. https://doi.org/10.1083/jcb.202206067.
